# Preliminary Biological Evaluation of ^18^F-FBEM-Cys-Annexin V a Novel Apoptosis Imaging Agent

**DOI:** 10.3390/molecules20034902

**Published:** 2015-03-17

**Authors:** Chunxiong Lu, Quanfu Jiang, Minjin Hu, Cheng Tan, Huixin Yu, Zichun Hua

**Affiliations:** 1 Ministry of Health & Jiangsu Key Laboratory of Molecular Nuclear Medicine, Jiangsu Institute of Nuclear Medicine, Wuxi 214063, China; E-Mails: jiangquanfu@jsinm.org (Q.J.); tangcheng@jsinm.org (C.T.); yuhuixin@jsinm.org (H.Y.); 2 Jiangsu Target Pharma Laboratories Inc., Changzhou High-Tech Research Institute of Nanjing University, Changzhou 213164, China; E-Mail: huminj98@163.com; 3 The State Key Laboratory of Pharmaceutical Biotechnology, Nanjing University, Nanjing 210093, China

**Keywords:** Cys-Annexin V, site-specific labeling, ^18^F-FBEM, apoptosis imaging

## Abstract

A novel annexin V derivative (Cys-Annexin V) with a single cysteine residue at its C-terminal has been developed and successfully labeled site-specifically with ^18^F-FBEM. ^18^F-FBEM was synthesized by coupling ^18^F-fluorobenzoic acid (^18^F-FBA) with N-(2-aminoethyl)maleimide using optimized reaction conditions. The yield of ^18^F-FBEM-Cys-Annexin V was 71.5% ± 2.0% (*n* = 4, based on the starting ^18^F-FBEM, non-decay corrected). The radiochemical purity of ^18^F-FBEM-Cys-Annexin V was >95%. The specific radioactivities of ^18^F-FBEM and ^18^F-FBEM-Cys-Annexin V were >150 and 3.17 GBq/µmol, respectively. Like the 1st generation ^18^F-SFB-Annexin V, the novel ^18^F-FBEM-Cys-Annexin V mainly shows renal and to a lesser extent, hepatobiliary excretion in normal mice. In rat hepatic apoptosis models a 3.88 ± 0.05 (*n* = 4, 1 h) and 10.35 ± 0.08 (*n* = 4, 2 h) increase in hepatic uptake of ^18^F-FBEM-Cys-Annexin V compared to normal rats was observed after injection via the tail vein. The liver uptake ratio (treated/control) at 2 h p.i. as measured via microPET correlated with the ratio of apoptotic nuclei in liver observed using TUNEL histochemistry, indicating that the novel ^18^F-FBEM-Cys-Annexin V is a potential apoptosis imaging agent.

## 1. Introduction

Apoptosis plays an important role, not only in physiology but also in pathology [1,2]. Dysregulation of apoptosis is associated with many diseases such as cancer, autoimmunity and neurodegenerative disorders. Therefore, it has significant clinical value of the detection and quantification of apoptosis *in vivo* for diagnosis and assessment of therapeutic efficacy. One of the early charateristics of apoptosis is the externalization of the phospholipid phosphatidylserine (PS) at the cell membrane [3,4]. Annexin V, a 36-kDa human protein, shows Ca^2+^-dependent binding to negatively charged phospholipid surfaces and was discovered as a vascular anticoagulant protein [5,6]. The anticoagulant activity is based on the high-affinity for PS. These characteristics make annexin V derivatives suitable candidates for imaging of apoptosis. Several annexin V tagged with bifunctional chelators (BFC) have been labeled with ^99m^Tc for single photon emission computed tomography (SPECT) imaging of apoptosis *in vivo* [7,8,9,10,11]. However, conjugation of BFC to annexin V for labeling with ^99m^Tc is usually done by targeting an amino group of one of the 21 lysine residues using BFC, but this method is rather non-specific as any of the –NH_2_ groups could be targeted. Recent studies have revealed that after structural modification in the recombinant expression annexin V can be directly marked with ^99m^Tc, giving derivatives such as ^99m^Tc(CO)_3_-HIS-cys-Anx V [12], ^99m^Tc-annexin V-117 [13] and ^99m^Tc-His10-annexin V [14]. These new annexin V molecules labeled by site-specific methods greatly improve sensitivity for detecting cell death *in vivo* [15]. Our group has reported a site-specific ^99m^Tc labeling method of a novel annexin V derivative (Cys-Annexin V) with a single cysteine residue at C-terminal [16]. ^99m^Tc-Cys-Annexin V is a potential SPECT imaging agent of apoptosis. However, because of its higher sensitivity, better spatial resolution and quantification properties a positron emission tomography (PET) analog would be very desirable. Some groups have reported the labeling of annexin V with N-succinimidy-4-^18^F-fluorobenzoate (^18^F-SFB) for PET imaging of apoptosis [17,18,19], however this labeling method is non-specific, as the ^18^F-SFB reacts with any available NH_2_ group in the protein. Thiol-reactive agents such as N-substituted maleimides can be used to modify proteins on the cysteine group [20]. ^18^F-N-[2-(4-Fluoro-benzamido)ethyl]maleimide (^18^F-FBEM) was used to label thiol-containing proteins as a novel site-specific labeling prosthetic group [21,22,23]. We report herein the labeling and preliminary *in vivo* evaluation of the novel ^18^F-FBEM-Cys-Annexin V in normal mice and in rat models of apoptosis induced by cycloheximide. In mice the tracer uptake was studied by dynamic microPET imaging and microPET in a rat model of hepatic apoptosis. Apoptosis was confirmed *in situ* on liver slices using the terminal deoxynucleotidyl transferase (TdT) dUTP nick end labeling (TUNEL) assay.

## 2. Results and Discussion

### 2.1. Radiolabeling

Annexin V has been labeled non-specifically with a number of isotopes, including ^99m^Tc, ^124^I, ^18^F and ^68^Ga [11,17,18,24,25]. Site-specific labeling of annexin V can help improve its sensitivity for detecting cell death *in vivo*. To take advantage of the higher resolution and more accurate quantification of PET, labeling annexin V with short half-life positron-emitters such as ^18^F is of particular interest. In this study site-specific labeling of Cys-Annexin V with ^18^F-FBEM as prosthetic group is presented.

**Figure 1:**
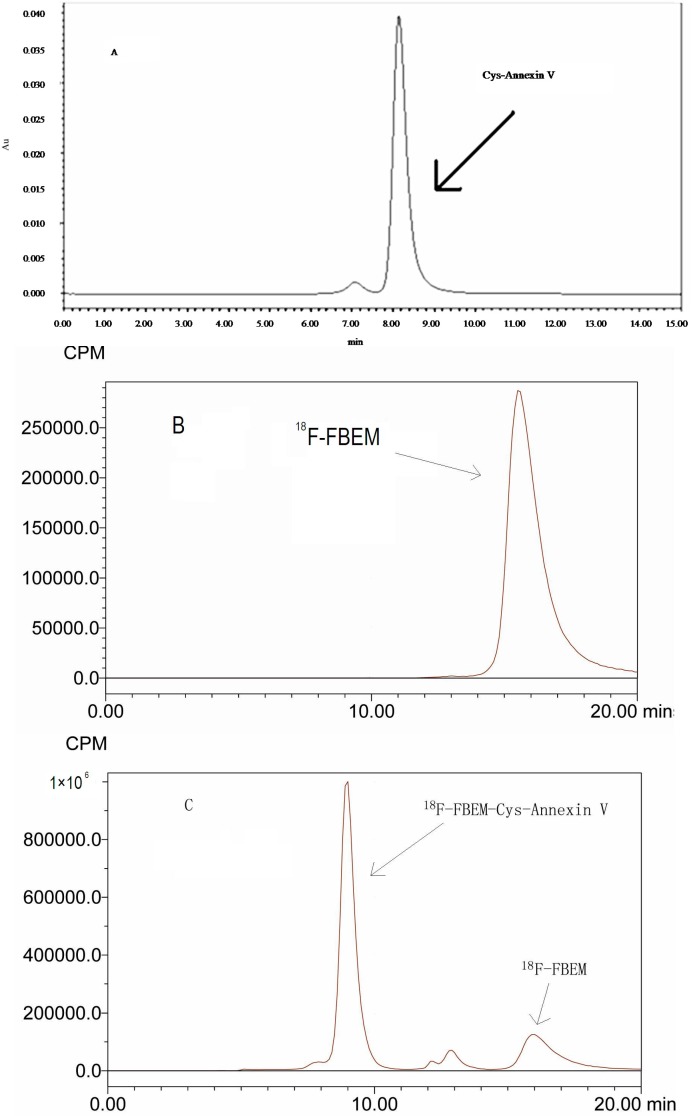

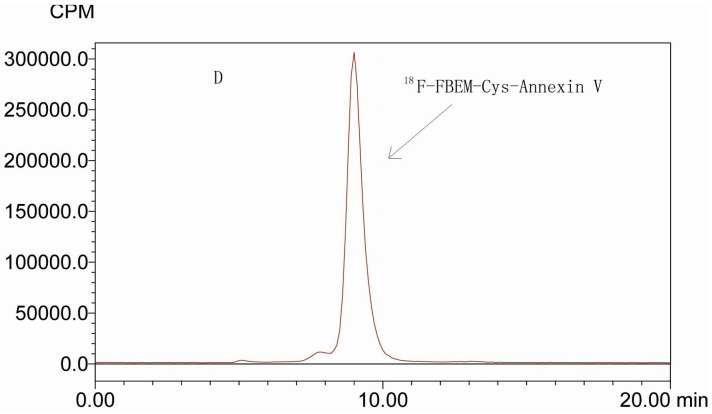
HPLC chromatogram (isocratic, 0.05 mol/L phosphate buffer (pH = 7.0), flow 0.8 mL/min) of: (**A**) Cys-Annexin V, t_R_ = 8.6 min (UV); HPLC radiochromatograms of (**B**) ^18^F-FBEM, t_R_ = 15.9 min, (**C**) reaction mixture (^18^F-FBEM-Cys-Annexin V, t_R_ = 9.1 min, ^18^F-FBEM, t_R_ = 15.9 min) and (**D**) ^18^F-FEBM-Cys-Annexin V, t_R_ = 9.1 min.

As see in Figure 1, Cys-Annexin V and ^18^F-FBEM-Cys-Annexin V were eluted at retention times of 8.6 min and 9.1 min, respectively, whereas ^18^F-FBEM eluted at a retention time of 15.9 min. According to HPLC analysis, the radiochemical purity of ^18^F-FBEM-Cys-Annexin V was above 95%. The total synthesis time for ^18^F-FBEM was about 100 min and 428 ± 65 MBq (*n* = 4) pure ^18^F-FBEM was obtained from 18.5 GBq ^18^F-fluoride. 81–262 MBq ^18^F-FBEM-Cys-Annexin V was obtained from 111–370 MBq ^18^F-FBEM as the radiochemical yield was 71.5% ± 2.0% (*n* = 4, based on the starting ^18^F-FBEM, non-decay corrected). The specific radioactivities of ^18^F-FBEM and ^18^F-FBEM-Cys-Annexin V were above 150 MBq/µmol and 3.17 GBq/µmol, respectively.

### 2.2. In Vitro Stability of ^18^F-FBEM-Cys-Annexin V

**Figure 2:**
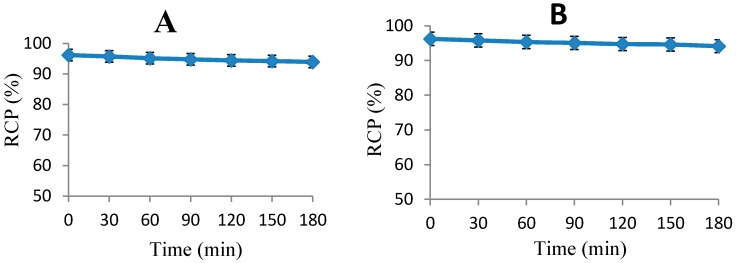
Stability of ^18^F-FBEM-Cys-Annexin V at different intervals in (**A**) PBS and (**B**) human serum.

To determine the radioactive decomposed side products which may accumulate in non-target organs, the stability of ^18^F-FBEM-Cys-Annexin V was studied. The results of the stability of ^18^F-FBEM-Cys-Annexin V in (A) phosphate buffered saline (PBS, 0.1 mol/L, pH 7.2) and (B) human serum, respectively, are presented in Figure 2. The results show that the ^18^F-FBEM-Cys-Annexin V is more stable during biodistribution and PET imaging studies.

### 2.3. Blood Kinetics Studies

**Figure 3:**
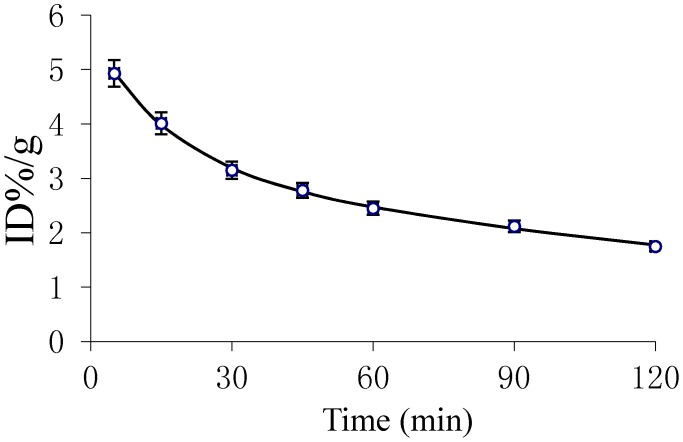
Pharmacokinetic curve for ^18^F-FEBM-Cys-Annexin V in mice.

Pharmacokinetic parameters, obtained using the DAS 2.1.1 pharmacokinetic calculation program, are listed in Table 1. Figure 3 shows the blood clearance of ^18^F-FEBM-Cys-Annexin V in the mice 2 h post-injection. Pharmacokinetics of ^18^F-FEBM-Cys-Annexin V comply with the two-compartment model with the pharmacokinetic equation of C = 2.359e^−0.0^^62t^ + 3.288e^−0.005t^ (where C is radiopharmaceutical activity (%ID/g) in blood, t is the time after injection). The half-life of distribution phase (t_1/2α_) and half-life of elimination phase (t_1/2β_) were 11.261 and 134.62 min, respectively, which showed that ^18^F-FBEM-Cys-Annexin V can be absorbed quickly and eliminated slowly. Biological availability was represented by area under concentration-time curve (AUC) and the values of clearance (CL) and AUC were 0.031 and 643, respectively.

**Table 1:** Pharmacokinetic parameters of the ^18^F-FEBM-Cys-Annexin V in mice.

Parameter (units)	^18^F-FEBM-Cys-Annexin V
K_12_ (min^−1^)	0.02
K_21_ (min^−1^)	0.038
K_e_ (min^−1^)	0.009
CL (%ID/g/min)	0.031
T_1/2α_ (min)	11.261
T_1/2β_ (min)	134.62
AUC (%ID/g·min)	634.123

In the early phase, the blood clearance of ^18^F-FEBM-Cys-Annexin V was slow. After 2 h, the radioactivity concentration of the tracer agent in blood reaches an equilibrium which coincides with the pharmacokinetic parameters CL, AUC and the pharmacokinetic curves.

### 2.4. Dynamic MicroPET Images of Normal ICR Mice

**Figure 4:**
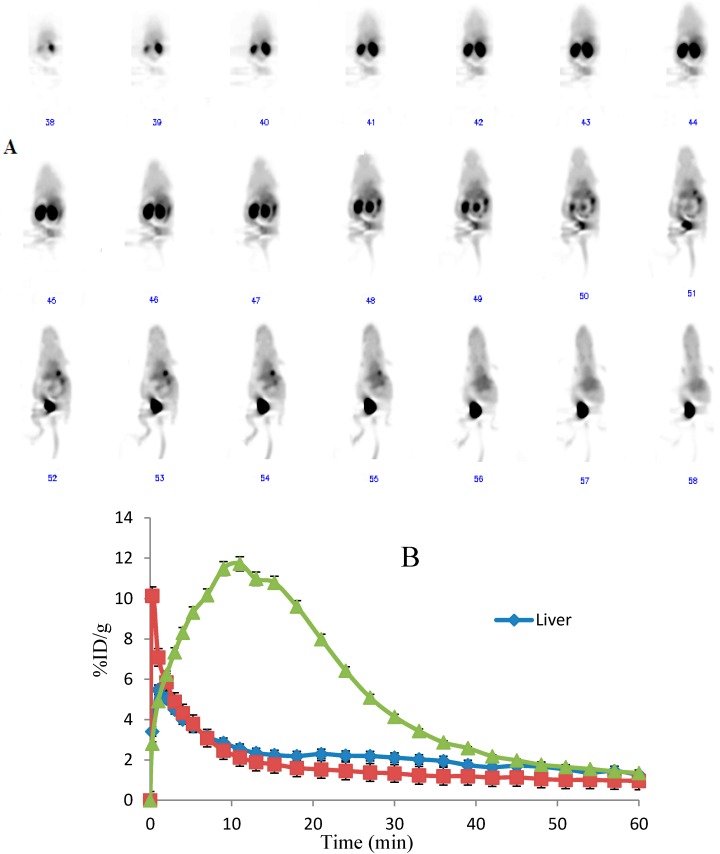
(**A**) Whole body coronal microPET images of ICR mouse from a 60 min dynamic scan after injection of 3.7 MBq ^18^F-FBEM-Cys-Annexin V. (**B**) Quantified time-activity curves of major organs (liver, heart and kidney) after injection of 3.7 MBq ^18^F-FBEM-Cys-Annexin V in normal ICR mice (*n* = 4).

Representative time-activity curves of the major organs (kidneys, liver and heart) were derived from 60-min dynamic microPET scans after intravenous administration of ^18^F-FBEM-Cys-Annexin V tracers (Figure 4). The radioactivity kinetics were calculated from a region-of interest analysis of the dynamic small animal PET scans over the heart (squares; mainly representing the cardiac blood pool), kidney (triangles) and liver (diamonds).^18^F-FBEM-Cys-Annexin V was excreted mainly through the kidneys, as evidenced by the higher renal uptake at early time points and excretion via the bladder. The kidney uptake reached a peak (11%ID/g) at 13 min after injection and then decreased to 1.43%ID/g at 60 min p.i.

### 2.5. Imaging of Rat Model of Apoptosis

**Figure 5:**
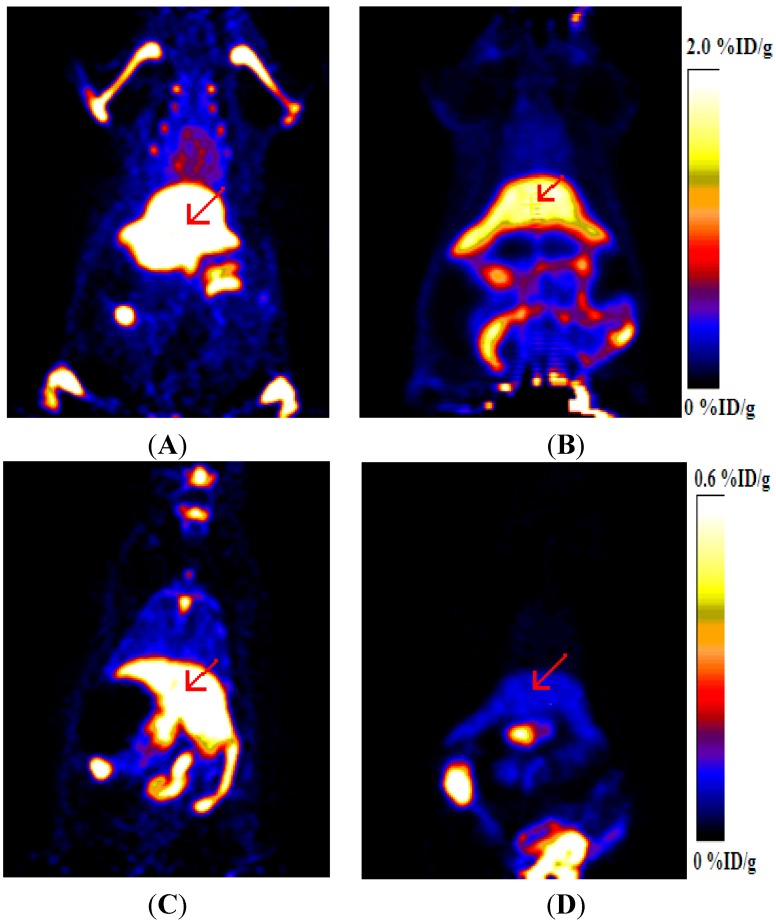
MicroPET images of CHX-treated and normal rats after injection of ^18^F-FBEM-Cys-Annexin V. (**A**) CHX-treated at 1 h p.i. (**B**) Normal at 1 h p.i. (**C**) CHX-treated at 2 h p.i. (**D**) Normal at 2 h p.i.

Four rats were treated with cycloheximide to induce liver apoptosis and two rats were used as the control group. Figure 5 shows the representative coronal microPET images of cycloheximide (CHX)-treated and normal rats at different times after intravenous injection of 8.2 MBq ^18^F-FBEM-Cys-Annexin V. ^18^F-FBEM-Cys-Annexin V tracer uptake in the liver (arrow) was increased with CHX treatment. The uptake ratios (treated/control) of liver were 3.88 ± 0.05 (*n* = 4) and 10.35 ± 0.08 (*n* = 4), respectively, at 1 h and 2 h p.i. 

**Figure 6:**
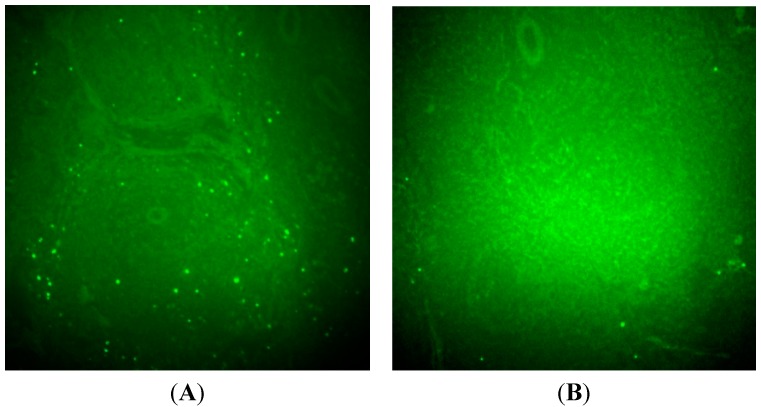
Representative TUNEL-stained images of liver specimen in CHX-treated rats (**A**), control rats (**B**). Green color dot represents positive TUNEL staining.

There were no differences in the blood pool activity between treated and control rats. TUNEL–staining images of liver sections were shown in Figure 6. These images show that the number of apoptotic nuclei in CHX-treated liver was more compared to that in non-treatment control rats. The uptake ratio (treated/control) of liver at 2 h p.i. as measured via microPET correlated with the ratio (treated/control) of apoptotic nuclei in liver observed using TUNEL histochemistry.

## 3. Experimental Section

### 3.1. General Information

Unless otherwise specified, all reagents were of analytical grade and were obtained from commercial sources. Cys-Annexin V was supplied by Jiangsu Target Pharma Laboratories Inc. (Changzhou, China). ^18^F fluoride was obtained from a cyclotron (HM67, Sumitomo Heavy Industries, Ltd, Tokyo, Japan) located at the Jiangsu Institute of Nuclear Medicine by proton irradiation of ^18^O-enriched water. A Waters high-performance liquid chromatography (HPLC) system (Waters, Milford, MA, USA) equipped with a Waters 2998 photodiode array detector (PDA) and a semi-preparative C18 HPLC column (250 × 10 mm, 5 μm, CHROM-MATRIX BIO-TECH) was used for ^18^F-FBEM purification. The flow rate was 3 mL/min, and the mobile phase changed from 95% solvent A (0.1% trifluoroacetic acid in water) and 5% solvent B (0.1% trifluoroacetic acid in acetonitrile) (0–2 min). The mobile phase was gradually changed to 35% solvent A and 65% solvent B at 32 min. The UV absorbance was monitored at 218 nm, and the UV spectrum was checked with the PDA detector.

Analytical HPLC was performed on Waters Breeze system with a TSK-GEL column (swG2000SWXL, 300 × 7.8 mm 5 µm, Tosoh Bioscience Co., Ltd, Shanghai, China). The absorbance was measured on the UV detector at 278 nm. Radioanalysis of the labeled compound was conducted using a Cd (Te) detector. The flow rate was adjusted to 0.8 mL/min and the isocratic mobile phase was 0.05 mol/L phosphate buffer (pH = 7.0).

A microPET system (Inveon, Siemens Co. Knoxville, TN, USA) and a fluorescence microscope (X51, Olympus, Tokyo, Japan) were used. The animal experiments in this study were approved by the Animal Care and Ethnics Committee of Jiangsu Institute of Nuclear Medicine.

### 3.2. Preparation of ^18^F-N-[2-(4-Fluorobenzamido)ethyl]maleimide

^18^F-N-[2-(4-Fluorobenzamido)ethyl]maleimide (^18^F-FBEM) was prepared as previously described using a semi-automatic method with some modifications [22,26]. Briefly, the precursor, ethyl 4-(trimethylammonium) benzoate trifluoromethanesulfonic acid salt (10 mg, 12 µmol) in anhydrous acetonitrile (1.0 mL) was heated at 100 °C for 10 min in a sealed vial with 18.5 GBq [^18^F]fluoride in the presence of dried Kryptofix2.2.2 (15 mg 6 µmol) and K_2_CO_3_ (5 mg, 3.6 mmol). The intermediate was hydrolyzed with NaOH (0.5 M, 0.5 mL) at 90 °C for 5 min. After acidification with 7.5 mL 0.1 M HCl, the solution was loaded onto an activated C18 Sep-Pak column (Waters). The cartridge was then eluted with 3 mL acetonitrile and the eluate was subsequently evaporated at room temperature with a stream of nitrogen to obtain ^18^F-fluorobenzoic acid (^18^F-FBA). ^18^F-FBA was treated with N-(2-aminoethyl)maleimide (MAL, 15 mg, 59 µmol), diethyl cyanophosphonate (20 µL, 99 µmol), and N,N-diisopropylethylamine (40 µL, 240 µmol) in anhydrous acetonitrile (0.5 mL). The resulting solution was heated at 75 °C for 7 min. The reaction was quenched by adding water (8.5 mL) and loaded onto an activated C18 Sep-Pak column. The cartridge was eluted with 1 mL ethanol which was then loaded on to the semi-preparative HPLC. The radioactive peak eluting at ~18 min was collected and passed through a C18 Sep-Pak column which was activated by EtOH/water. The cartridge was washed with 20 mL water and then eluted with 1 mL CH_2_Cl_2_. The organic layer was evaporated to dryness at room temperature under a stream of nitrogen and utilized for further Cys-Annexin V labeling. The total synthesis time for ^18^F-FBEM was about 100 min and 428 ± 65 MBq (*n* = 4) radiochemically pure ^18^F-FBEM was obtained from 18.5 GBq ^18^F-fluoride.

### 3.3. Preparation of ^18^F-FBEM-Cys-Annexin V

**Scheme 1:**
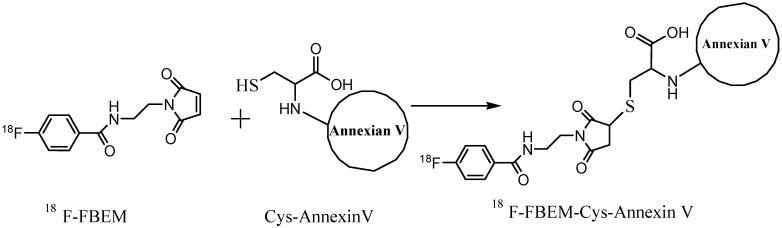
Syntheses of ^18^F-FBEM-Cys-Annexin V.

The isolated ^18^F-FBEM (111–370 MBq) in 10 µL of ethanol was added to a solution of Cys-Annexin V (50–100 µg in 100 µL, pH = 7.2) PBS (Scheme 1), and the mixture was allowed to react at room temperature for 15–30 min and loaded onto a NAP-5 column (GE Healthcare, Buckinghamshire, UK). The NAP-5 column was eluted with 250 µL portions of PBS. The most concentrated fraction containing the radiolabeled protein (fraction 3, 81–262 MBq) was collected and used for the biological experiments.

### 3.4. In Vitro Stability of ^18^F-FBEM-Cys-Annexin V

The *in vitro* stabilities of freshly prepared ^18^F-FBEM-Cys-Annexin V were performed in PBS (0.1 mol/L, pH 7.2) and human serum, respectively, for different time intervals (0–6 h) at 37 °C in a water bath.)

### 3.5. Blood Kinetics Studies of ^18^F-FBEM-Cys-Annexin V in Normal Mice

Five ICR mice were injected via the tail vein with ^18^F-FBEM-Cys-Annexin V (0.2 mL) and activity of approximately 3.7 MBq. Ten μL of blood were taken from tails at 5, 15, 30, 45, 60, 90 and 120 min after injection. The activity for each sample was determined by a γ-counter and expressed as percentage of injection dose per gram (%ID/g).

### 3.6. Dynamical MicroPET Images of Normal Mice

Four ICR mice were anesthetized with 1%–2% isoflurane, positioned prone, immobilized, and injected via the tail vein with 3.7 MBq ^18^F-FBEM-Cys-Annexin V (0.2 mL) and imaged dynamically for 1 h. The images were reconstructed using a two dimensional ordered-subset expectation maximization (2D OSEM) algorithm without correction for attenuation or scattering. For each scan, regions of interest (ROIs) were drawn over the liver and major organs using the vendor-supplied software (ASI Pro 5.2.4.0) on decay-corrected whole-body coronal images. The radioactivity concentrations (accumulation) within the liver, heart and kidneys were obtained from mean pixel values within the multiple ROI volume and then converted to megabecquerel per milliliter per minute using the calibration factor determined for the Inveon PET system. These values were then divided by the administered activity to obtain (assuming a tissue density of 1 g/mL) an image-ROI-derived percent injected dose per gram (%ID/g).

### 3.7. MicroPET Images of Rat Model of Apoptosis

Four male SD rats (258 ± 2 g) were treated IV with 10 mg/kg cycloheximide to induce liver apoptosis. Two male SD rats (262 g and 256 g) were treated IV with saline as the control group. 3 h after treatment, the rats were anesthetized with 1%–2% isoflurane and were injected via the tail vein with 8.2 MBq ^18^F-FBEM-Cys-Annexin V (0.2 mL). Ten-minute static scans were acquired at 1 and 2 h after injection with a MicroPET (Inveon, Siemens), respectively, which was from 1 h to 1 h and 10 min or from 2 h to 2 h and 10 min. Immediately after MicroPET imaging, the livers were dissected. Then, using the livers, formalin-fixed paraffin-embedded specimens were prepared for Terminal deoxynucleotidyl transferase-mediated nick end labeling (TUNEL) staining.

### 3.8. TUNEL Staining

Because our imaging studies were designed to determine the uptake and biodistribution of ^18^F-FBEM-Cys-AnnexinV after chemically induced apoptosis, it was important to confirm apoptosis in the livers of treated rats by independent methods that provide quantitative results. A marker of apoptosis was scored by performing a TUNEL assay that measures DNA fragmentation, a characteristic feature of apoptosis. Terminal deoxynucleotide transferase adds labeled nucleotides to the 3' termini at double-stranded breaks in the fragmented DNA. TUNEL assays were performed according to the manufacturer’s instructions, using the fluorescein-conjugated Colorimetric TUNEL Apoptosis Assay Kit (Beyotime Institute of Biotechnology, Shanghai, China). Briefly, slices were freed of paraffin through xylene and graded EtOH washes and then incubated with proteinase K (Beyotime Institute of Biotechnology, 2 mg/mL in 10 mmol/L Tris, pH 8.0). After proteinase digestion, the slides were equilibrated in pH 7.4 buffer, the terminal deoxynucleotidetransferase enzyme and Biotin-dUTP labeling mix (Beyotime Institute of Biotechnology) were added, and the slides were incubated at 37 °C for 1 h in a humid chamber. The number of TUNEL-positive cells was counted on 10 randomly selected ×100 fields for each section by use of an Olympus fluorescence microscope.

## 4. Conclusions

Cys-annexin V, a novel annexinVderivative with a single cysteine residue at the C-terminal, could be site-specifically labeled with ^18^F-FBEM in high yields and high radiochemical purity. In normal mice, ^18^F-FBEM-Cys-Annexin V was excreted mainly through the renal pathway. Hepatic uptake of ^18^F-FBEM-Cys-Annexin V was significantly increased in the rats treated with CHX compared to controls, which correlated well with the increase in cell death observed using TUNEL histochemistry. These results indicate that the novel ^18^F-FBEM-Cys-Annexin V is a potential apoptosis imaging agent and further study is needed.
